# Sample size estimation for local hypothesis testing of functional data in medical studies: method comparison, recommendations, and a web application

**DOI:** 10.1186/s12874-026-02772-w

**Published:** 2026-01-22

**Authors:** Mohammad Reza Seydi, Johan Strandberg, Todd C. Pataky, Lina Schelin

**Affiliations:** 1 Department of Statistics, Umeå School of Business and Economics, Umeå University, Umeå, Sweden; 2 Department of Human Health Sciences, Kyoto University Graduate School of Medicine, Kyoto, Japan

**Keywords:** Functional data analysis, Hypothesis test, Power analysis, Sample size, Statistical power

## Abstract

**Background:**

Recent medical studies have shown an increasing interest in inferential methods for analysing functional data, while statistical power analysis for sample size planning for such data is less explored. As a result, researchers often rely on classical scalar approaches to estimate sample size, despite working with functional data. This can substantially underestimate the required sample sizes. Moreover, there are no guidelines to assist researchers in planning, conducting, and reporting sample size estimation for studies analysing functional data.

**Methods:**

Two functional data sets from medical sciences are used in a simulation study to explore a functional approach for sample size planning. These data represent two distinct patterns in mean function differences. Six well-known local inferential methods are evaluated for two-population comparisons of functional data. The evaluation focuses on the sample sizes required to achieve the target statistical power, under different data characteristics and assuming equal group sizes and stationary noise in the data generation process. We have also developed an interactive web-based application that helps researchers in performing a priori power analysis by allowing them to explore how changes in data characteristics affect statistical power, and consequently, the required sample size.

**Results:**

Our comparison revealed distinct patterns in the estimated sample sizes for different data characteristics and inferential methods. Even when based on the same baseline data, the required sample sizes to achieve a target statistical power of 0.80 differed noticeably, ranging from very small to moderately large sample sizes, depending on the mean function pattern, underlying noise characteristics, and inferential approach.

**Conclusions:**

Overall, our results emphasise the importance of appropriate sample size planning and inferential method selection for valid inference in medical studies that include functional data analysis. Based on these findings, we provide guidance for researchers to follow, from study design conception through to reporting.

**Supplementary Information:**

The online version contains supplementary material available at 10.1186/s12874-026-02772-w.

## Background

Functional data are collected in many medical studies, and may appear as dense longitudinal data or waveforms [1]. For instance, continuous glucose monitoring produces glucose curves used in diabetes research [2], and biomechanical trajectories, such as ground reaction forces and joint moments, are widely used in orthopaedic and rehabilitation studies to assess injury risk or treatment response [3]. As these data are increasingly collected, interest in functional data analysis has been growing in medicine [4], biomechanics [5, 6], and human movement sciences [7]. Consequently, hypothesis testing for functional data has also become common in the medical and biomedical fields. Comparison of plasma glucose curves for different categories of body mass index [8], and comparison of growth trajectories between normal and low birth-weight infants [9] are two examples within these fields.

Despite this increasing interest in the analysis of functional data and recent developments [10], approaches related to the data collection process have not received much attention. One specific aspect is statistical power analysis for sample size estimation, which is typically requested in applications for ethical approval and in author guidelines for peer-reviewed journals [11]. A priori power estimation is essential because it determines the appropriate sample size needed to ensure that studies are sufficiently powered to detect meaningful effects, avoiding underpowered studies that could fail to identify true effects. Proper power analysis helps prevent excessive data collection in medical studies, minimising wasted time and resources while reducing the risk of misinterpretation. Although performing a power analysis prior to data collection is crucial, it has often been neglected and, in many cases, not reported at all [12, 13].

When sample size estimation is reported, a recurring issue is that sample sizes are often calculated using zero-dimensional data (scalar approaches), despite the analyses involving functional data [14, 15]. Power-relevant conclusions in these studies might change if sample sizes were estimated using a functional approach rather than a zero-dimensional approach. It has been demonstrated that the sample size required to achieve a target level of statistical power for functional data is considerably larger than that needed for zero-dimensional data in some scenarios [12].

Hypothesis testing for functional data can be categorised into two approaches, global and local testing. Global hypothesis tests evaluate a single null hypothesis against an alternative over the entire domain of functional observation. A variety of global testing procedures have been proposed in the literature. For instance, Cuevas et al. [16] introduced a functional analysis of variance test to assess the equality of mean functions among multiple samples, and Shen and Faraway [17] developed a global functional F-test for model selection in functional linear models. A classical and naive approach, based on the $\mathcal {L}^2$ norm and bootstrap procedure, to test the equality of two population mean functions was introduced by Zhang et al. [18]. More recently, Koner and Luo [19] proposed a global projection-based method designed to detect group differences under sparse longitudinal designs. A modern non-parametric test introduced by Pomann et al. [20] compares the global underlying distributions of two functional samples. Despite their methodological differences, all of these global approaches generally focus on an overall decision and provide limited insights about the specific regions of the domain where differences may occur. In contrast, local hypothesis testing methods are designed to identify the particular intervals, time points or general subregions of the domain where differences occur. In this paper, we focus on local hypothesis testing for functional data.

Sample size estimation for functional data was described by Pataky et al. [21] based on the statistical parametric mapping (SPM), a local inferential method widely used in biomechanical experiments. Moreover, Pataky et al. [22] explored the region of interest and its consequence on the statistical power using the SPM. The Power1D toolbox implemented and developed by Pataky [23] is a framework to perform power calculation based on random field theory (RFT) and SPM, where the user has numerous options to generate arbitrary signals and noises. A comprehensive study on sample size estimation using SPM, with applications on locomotion kinematics and electromyography, was conducted by Luciano et al. [24]. This study analysed the relation between the amplitude and full-width-at-half-maximum (FWHM) of Gaussian signals and regions of interest on statistical power for fixed noise smoothness levels. The effect of the standard deviation of noise functions and various types of signals on the statistical power using the SPM method was investigated in Robinson et al. [12]. Despite the focus of the mentioned articles on sample size estimation using SPM, a variety of methods from the functional data analysis (FDA) literature have been commonly applied to functional data and research on sample size estimation for these methods remains largely absent.

New local non-parametric inferential methods for functional data are briefly introduced and compared by Mrkvička and Myllymäki [25]. The compared local methods include, e.g., the interval-wise testing procedure (IWT) [26], threshold-wise testing (TWT) [27], global envelope tests [28, 29], and iterative adaptive two-stage envelope [30]. Another detailed comparison of IWT, SPM, the non-parametric version of SPM, and a Benjamini-Hochberg procedure is found in Pataky et al. [31]. However, neither of these comparisons focused on power analysis and sample size estimation.

The aim of this paper is to provide guidance for researchers conducting a priori sample size estimation for functional data, with a particular focus on local functional two-sample tests, a common scenario in medical studies. Such guidance should improve study design, ethical review statements, and the interpretability of findings in medical studies. To reach this aim, we compared six widely used local methods in terms of the sample size needed to ensure valid inference. In a simulation study, we examined how the standard deviation and smoothness of noise functions, under varying data characteristics, influenced sample size estimation and FWER for all six methods. We selected vertical ground reaction force and knee joint moment trajectories as the baseline for our simulation study. These types of trajectory data are well-established in orthopaedic and rehabilitation research and exhibit functional characteristics relevant to our study. Although sample size estimation is based on achieving a desired level of statistical power, this criterion is not sufficient to fully assess the performance of inferential methods. Therefore, we also evaluated the family-wise error rate (FWER) to ensure that each method provides adequate error control.

In addition, we developed a web-based application using the R Shiny framework to facilitate power estimation with different methods. Users can explore the effects of parameters on publicly available datasets, pilot data or even by drawing data they expect to see. Overall, this study offers valuable information to help researchers determine the required sample size and select the most suitable approach for their specific data.

The rest of this article is organised as follows. Section 2 defines the functional hypothesis of interest and statistical power in this context, and gives a brief overview of the six local inferential methods employed in this study. Furthermore, this section describes the design of our simulation studies and provides a user guide for the Web application, detailing the contents of each tab and the application’s features. Section 3 presents the results of the simulation studies. Section 4 discuss the results, addresses limitations, and suggests possible directions for future research, while Sect. 5 contains concluding thoughts. Finally, Sect. 6 translates the results into practical recommendations.

## Methods

Assume two independent random samples $\{y_{1q}\}_{q=1,2,\ldots ,n_1}$ and $\{y_{2q}\}_{q=1,2,\ldots ,n_2}$ observed from two populations. For each sample, the sets $\{y_{11}(t),\ldots ,y_{1n_1}(t)\}$ and $\{y_{21}(t),\ldots ,y_{2n_2}(t)\}$ represent $n_1$ and $n_2$ continuous functions over $t \in \mathcal {D}$, respectively. Let $\hat{\mu }_1(t)$ and $\hat{\mu }_2(t)$ be the mean functions of the two samples and $\mu _1(t)$ and $\mu _2(t)$ denote the population mean functions. A local hypothesis test for functional data is defined as

$$\begin{aligned} \left\{ \begin{array}{ll} H_0^\mathcal {T} & : \forall t \in \mathcal {T} \subseteq \mathcal {D}, \quad \mu ^\mathcal {T}_1(t) = \mu ^\mathcal {T}_2(t)\\ H_1^\mathcal {T} & : \exists t \in \mathcal {T} \subseteq \mathcal {D} \quad \text {s.t.} \quad \mu ^\mathcal {T}_1(t) \ne \mu ^\mathcal {T}_2(t), \end{array}\right. \end{aligned}$$

where $\mathcal {T}$ is a subset or subsets of $\mathcal {D}$. Additionally, $\mu ^\mathcal {T}_1(t)$ and $\mu ^\mathcal {T}_2(t)$ denote restricted mean functions over $\mathcal {T}$. A local inferential method, which is designed to address the local hypothesis tests, can identify regions of the domain where the two population mean functions differ significantly. Details on the differences between local and global hypotheses can be found in Appendix A.

### Statistical Power

Statistical power is defined as the probability of rejecting a null hypothesis when it is false, i.e. when data are generated under the alternative hypothesis. Different terms for statistical power have been established for functional (curve) data by Pataky et al. [21], including omnibus power, which refers to the probability of rejecting the null hypothesis at least once within the domain $\mathcal {D}$ when data are generated under an alternative hypothesis. Additionally, centre or region of interest power indicates the probability of rejection within a predefined radius or region surrounding a specific point, while point of interest power describes the probability of rejection at a single point. This paper focuses exclusively on omnibus power.

To estimate statistical power in practice, data are generated under the alternative hypothesis where two population mean functions differ over at least a subset of the domain Afterwards, the inferential methods are applied to determine whether the null hypothesis is rejected at any part in the domain. For methods that provide *p*-value functions, this means checking whether this function falls below the significance level $\alpha$ at any region of the domain. For envelope-based methods, if the observed test statistic function falls outside the envelope at any portion in the domain, the null hypothesis is rejected at the given significance level. By repeating this procedure over many replications, the statistical power is estimated.

### Inferential methods

This study investigates six widely used local inferential methods for analysing functional or curve data (Table 1). Five of these methods control the FWER, which is the probability of making at least one Type I error (rejecting the null hypothesis incorrectly). One method controls the false discovery rate (FDR), defined as the expected proportion of Type I errors over all rejected hypotheses. Each method is briefly introduced below. The detailed descriptions of each method, including key assumptions and a step-by-step procedure to calculate the p-values function, are provided in Appendix B.

**Table 1:** Comparison of six local inference methods: Statistical Parametric Mapping (SPM), F-max, Interval-wise Testing (IWT), Threshold-wise Testing (TWT), Extreme Rank Length (ERL) Envelope, and Iterative Adaptive Two-stage Envelope (IATSE). The methods differ in terms of controlling family-wise error rate (FWER) or false discovery rate (FDR) and inference basis, such as permutation-based or using Euler characteristic (EC) densities. For further details on performance time, see Appendix C

Method	Control	Inference basis	Performance time
SPM	FWER	EC density	Fast
F-max	FWER	Permutation	Moderate
IWT	FWER	Permutation	Slow
TWT	FWER	Permutation	Moderate
ERL	FWER	Permutation	Moderate
IATSE	FDR	Permutation	Moderate

#### Statistical parametric mapping (SPM)

SPM was primarily developed by Friston et al. [32] to analyse time series of 3D functional magnetic resonance imaging. However, it has now been applied to data of various dimensionalities, including one-dimensional biomechanical functional (curve) data [33]. Using Gaussian RFT, SPM provides parametric solutions based on the expected Euler characteristic (EC) for functional hypothesis tests. SPM framework can be easily extended to different test statistics by appropriately modifying the EC densities.

#### F-max

F-max is a permutation-based method to address functional hypotheses with control over the FWER. By repeatedly shuffling group labels and recomputing the maximum statistic, it builds an empirical null distribution of global maxima. This method is commonly referred to as statistical non-parametric mapping (SnPM) in the RFT literature and was introduced by Holmes et al. [34]. Although this approach is slower than SPM, it relaxes the Gaussian assumption and does not need the distributional assumptions over Euler characteristic densities.

#### Interval-wise testing (IWT)

The IWT, proposed by Pini and Vantini [26], is a local inferential method designed to address functional hypotheses. This method partitions the domain into subintervals and performs a permutation-based test on each combined interval using the integrated test statistic. Then, it aggregates evidence across combined subintervals to provide interval-wise control of FWER over the continuum. Since IWT simultaneously considers almost every possible interval, it is computationally intensive.

#### Threshold-wise testing (TWT)

The TWT, introduced by Abramowicz et al. [27], is another local inferential method designed to address functional hypotheses and controls FWER. This method does not use a predefined family like IWT, therefore, it is a data-dependent technique based on permutation. TWT divides the domain into equally sized segments and estimates the *p*-value function using a set of thresholds. Unlike other methods that rely on point-wise test statistics, the TWT and IWT approaches utilise integral test statistics over a collection of subsets.

#### Extreme rank length (ERL) envelope

Global envelope tests were introduced and developed by Myllymäki et al. [28] for spatial processes. A functional hypothesis can also be tested using these envelopes [35]. The extreme rank length is one of the global envelope methods in which permuted test statistic functions are ordered so that functions with longer lengths of pointwise extreme ranks are deemed more extreme. This method provides control over the FWER. The ERL envelope method constructs simultaneous confidence bands (global envelopes) by ordering permuted test statistic functions according to their extremeness. The null hypothesis is rejected at a specified significance level if the observed test statistic function falls outside the envelope at any point in the domain.

#### Iterative adaptive two-stage envelope (IATSE)

IATSE is a permutation-based envelope test developed by Mrkvička and Myllymäki [30]. This method was designed for envelope testing to control the FDR rather than the FWER. IATSE iteratively refines an envelope based on ranked permutations and an adaptive estimate of the proportion of true nulls. Using this approach, the null hypothesis is rejected if the observed test statistic falls outside the defined envelope. Among the methods that have control over the FDR, the iterative adaptive two-stage envelope is included in this study due to its marginally better performance with fewer permutations, as reported by Mrkvička and Myllymäki [25].

### Simulation study

This section summarises the simulation study used to evaluate the performance of the methods. The section begins by describing the data underlying the simulations and then provides details about the simulation setup.

#### Data description

**Fig. 1:**
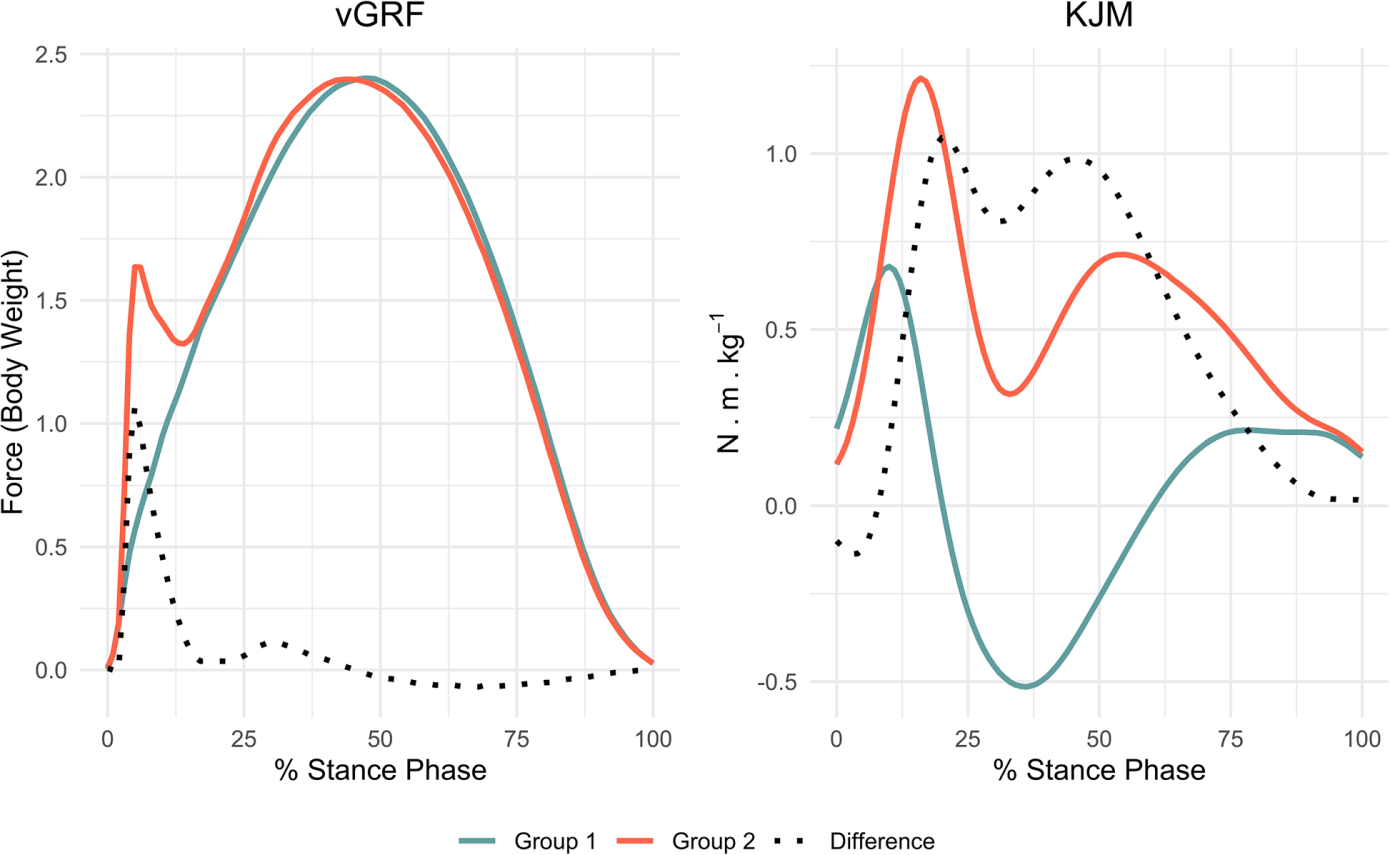
An illustration of the two publicly available datasets. The solid coloured lines represent the two groups’ mean functions while the dotted line is the difference between the two mean functions

The simulation study uses two publicly available biomechanical datasets (Fig. 1) that reflect functional data frequently encountered in orthopaedic and rehabilitation science. These datasets include force curves and joint moment trajectories, both of which capture movement-related patterns relevant to medical and biomechanical assessment. The first data set is the time and body weight normalised vertical ground reaction force (vGRF) from Phan et al. [36]. The vGRF data were collected to demonstrate that running quietly reduces peak ground reaction forces and vertical loading rate while altering foot-strike technique. The second data set is the external knee joint moments (KJM) in the frontal plane from Robinson et al. [37]. This data set originates from male Australian amateur football players who were free from self-reported joint disorders and prior orthopaedic surgery. These data were used to evaluate the impact of different knee modelling approaches on outcomes and on classification of anterior cruciate ligament (ACL) injury risk. The vGRF plot shows two mean functions of time and body weight normalised vertical ground reaction force during the stance phase of running under quiet sound (Group 1) and normal (Group 2) conditions (left panel of Fig. 1). The KJM plot displays the average functions of external knee joint moments in the frontal plane for inverse kinematic (Group 1) and direct kinematic (Group 2) (right panel of Fig. 1). These two data sets were selected because the differences between their mean functions exhibit distinct patterns, as can be seen in Fig. 1. The difference function for the vGRF data exhibits a substantial effect in a small region of the domain, while the KJM data show a substantial effect over a large part of the domain. These two scenarios allow us to assess the sensitivity of the methods, in terms of statistical power, to both localised and widespread effects. The simulations are conducted using only the mean curves. For illustrations of the complete data, the reader is referred to Phan et al. [36] and Robinson et al. [37].

#### Simulation setup

The sample functional (curve) data, $y_{iq}$ can be modelled as

$$\begin{aligned} y_{iq}(t)=\mu _{i}(t)+\epsilon _{iq}(t), \quad t \in \mathcal {D}, \end{aligned}$$

where the group is indicated by $i=1,2$, and the individual function is indicated by $q=1,...,n$ with *n* being the sample size for each group. The mean function for group *i* is represented by $\mu _{i}$, and $\epsilon _{iq}$ denotes the noise function of individual *q* for group *i*.

**Fig. 2:**
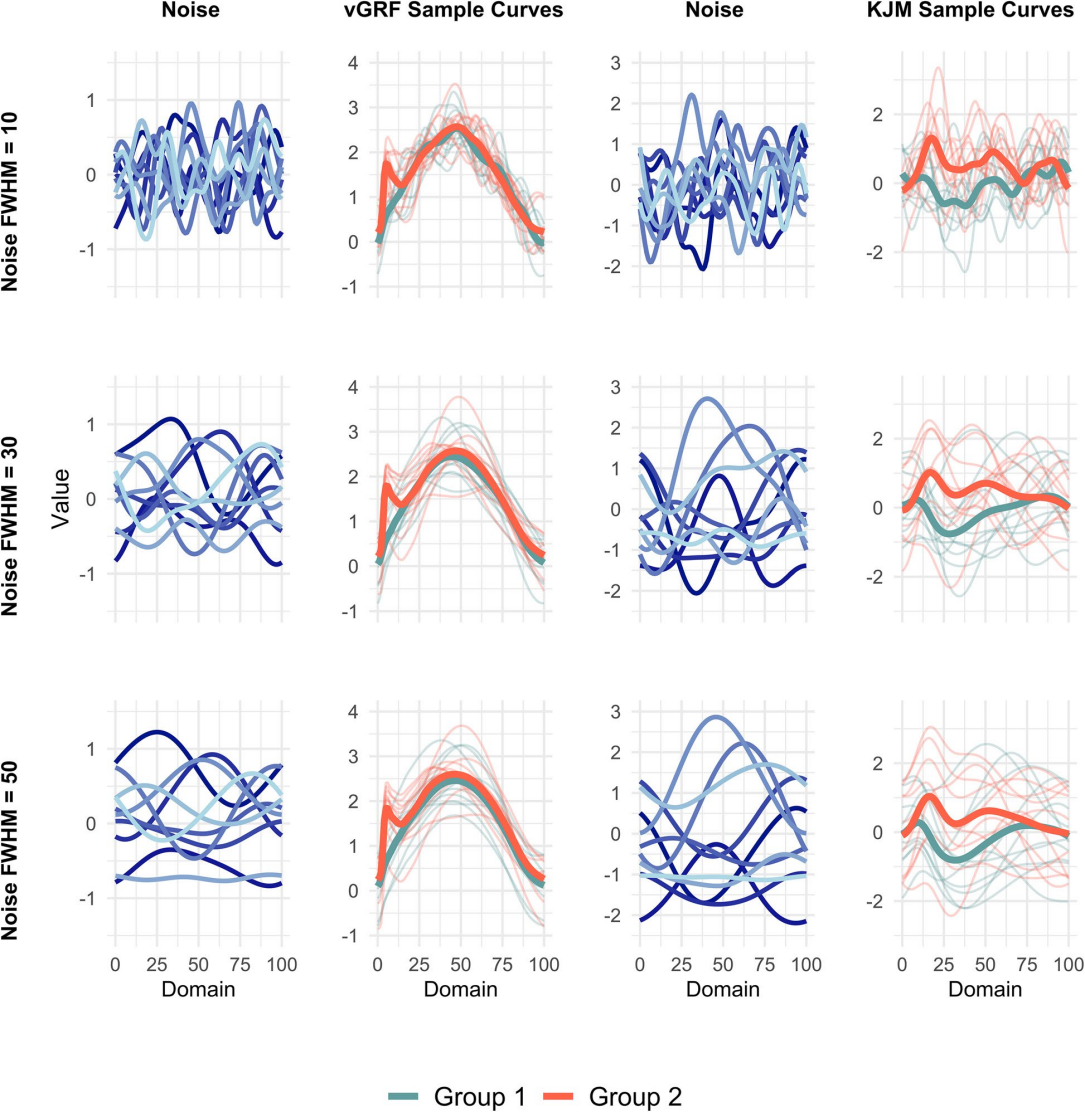
An illustration of generated noises and sample functions for different smoothness levels (Noise FWHM values). The sample size is 10, and the noise type is uncorrelated standard Gaussian. The noise standard deviation values in the first to last rows are 0.4, 0.5, and 0.6 for vGRF data and 0.8, 1 and 1.2 for KJM data

To simulate data, the mean functions of group 1 and group 2 (Fig. 1) for each dataset are denoted $\mu _{i}(t)$ for $i=1,2$ and $t \in \mathcal {D}$. Given that vGRF and KJM data are typically expressed as percentages of the stance phase, a domain length of $\mathcal {D}=[0,100]$ was selected. Uncorrelated Gaussian noise functions, $\epsilon _{iq}(t)$, with five different smoothness levels, Noise FWHM, are added to the mean functions to create $y_{iq}(t)$ (Fig. 2). The noise functions are set to be independent Gaussian errors $\epsilon _{iq}(t)\sim \mathcal {N}\bigl (0,\text {Noise SD}^2)$ for all $t \in \mathcal {D}$, where Noise SD is the constant noise standard deviation parameter used in the simulations ($\text {Noise SD}=\{0.4,0.5,0.6\}$ for the vGRF data and $\text {Noise SD}=\{0.8,1.0,1.2\}$ for the KJM data). These Noise SD values were selected to represent three different magnitudes of standard deviation: small, moderate, and large. Noise FWHM is defined as $\text {Noise FWHM} = 2\sigma \sqrt{2 \ln (2)}$, where $\sigma$ is the standard deviation of the Gaussian kernel which is used to smooth data. Five different levels were selected for the noise smoothness, $\text {Noise FWHM}=\{10, 20, 30, 40, 50\}$. This range of Noise FWHM values covers the smoothness levels commonly observed in experimental datasets in human movement science [38].

For each of the six methods described in Sect. 2, the minimum required sample size per group was estimated as the smallest size for which the confidence interval for statistical power includes the target value of 0.80, based on 2500 simulation runs. The confidence interval for the estimated statistical power is constructed using the binomial distribution. This procedure was repeated across all combinations of noise standard deviation and smoothness levels. A step-by-step algorithm describing the simulation procedure can be found in Algorithm 1. For all simulations, the domain was discretised using 101 points. All permutation-based methods were evaluated using 1000 permutations. The simulation code is available at https://github.com/mr-seydi/SampleSize_FDA.

**Figure:**
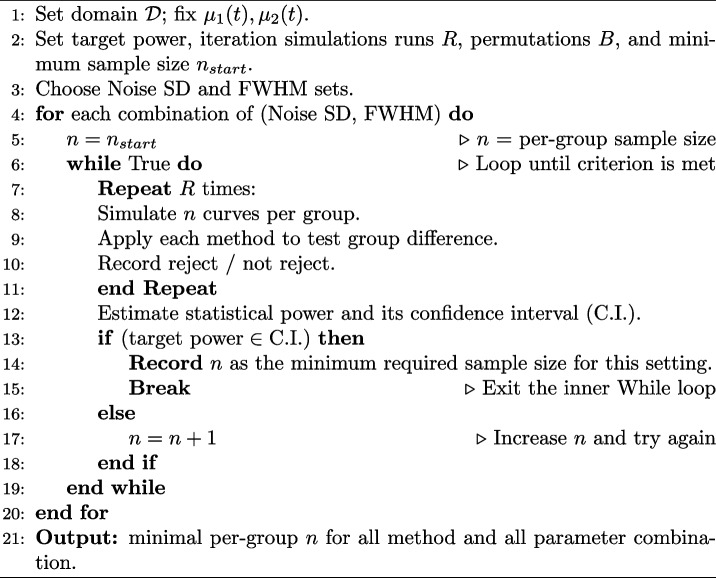
**Algorithm 1** Concise procedure for per-group sample size estimation

To evaluate the FWER, 2500 replicates of data are generated under the null hypothesis, assuming identical mean functions. Consequently, the simulation model includes only noise functions with predetermined smoothness and standard deviation. The sample sizes utilised in the model to generate the data are the estimated sample sizes required to achieve 0.80 statistical power, as reported in Fig. 4.

### Web application: Power ShinyApp

**Fig. 3:**
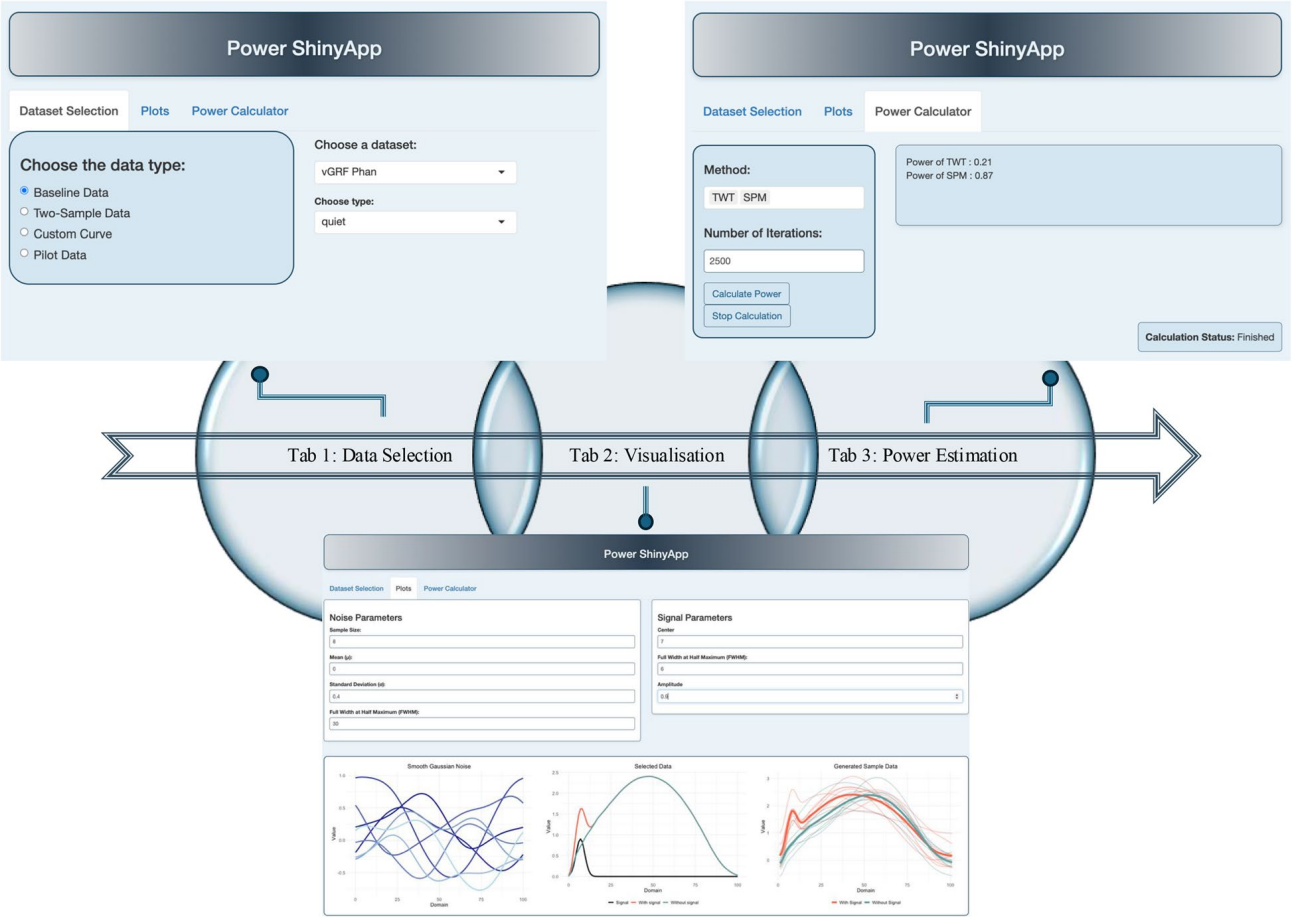
Roadmap of the Power ShinyApp, illustrating its three main tabs

The Power ShinyApp (https://github.com/mr-seydi/PowerShinyApp) is a web-based interactive application developed using R’s Shiny framework [39]. The developed app is designed to perform statistical power analysis for functional data using multiple statistical methods. The application supports multiple data inputs, interactive visualisation, and statistical power computation to provide a flexible tool for simulation-based power analysis (Fig. 3).

The current implementation of the Power ShinyApp operates under several key assumptions that should be noted. Specifically, the omnibus power has been used as the definition of statistical power. The power calculations assume equal group sizes. Furthermore, the simulated functional data relies on a stationary noise structure. The application currently restricts the noise component to Gaussian noise only, which is then smoothed using a Gaussian kernel. Users should be aware of these assumptions when interpreting the results of the power analysis.

#### Tab 1: data selection

Users can select datasets that represent the mean functions (curves) of two groups from various publicly available biomechanical datasets. As an alternative, users can draw custom mean functions directly on a coordinate plane. In this mode, the user can control the smoothing of the drawn function using a LOESS regression [40] with a tunable smoothness parameter. Users may also select one group mean function from the mentioned datasets and add a Gaussian pulse to create the other group mean function. They may also upload their pilot data to perform power analysis. This option allows users to perform a statistical power analysis using estimated parameters from pilot data.

#### Tab 2: visualisation

The application provides visualisations of noise functions, population mean functions, and a random illustration of the sampled functional data. The noise plot displays the smoothed Gaussian noise generated with user-defined parameters such as sample size, mean, Noise SD, and Noise FWHM. The two-group mean plot shows the mean functions for the two populations. When one group mean function is selected, or one custom function is drawn, the pulse parameters (centre, amplitude, and signal FWHM) are adjustable, enabling the user to explore the effects of different signal characteristics.

#### Tab 3: power estimation

Users can select one or more of the inferential methods mentioned in this paper, start the power computation, or cancel it if needed. The estimated statistical power upon completion is displayed in the app. It should be noted that some methods are computationally intensive and require more time to execute.

## Results

**Fig. 4:**
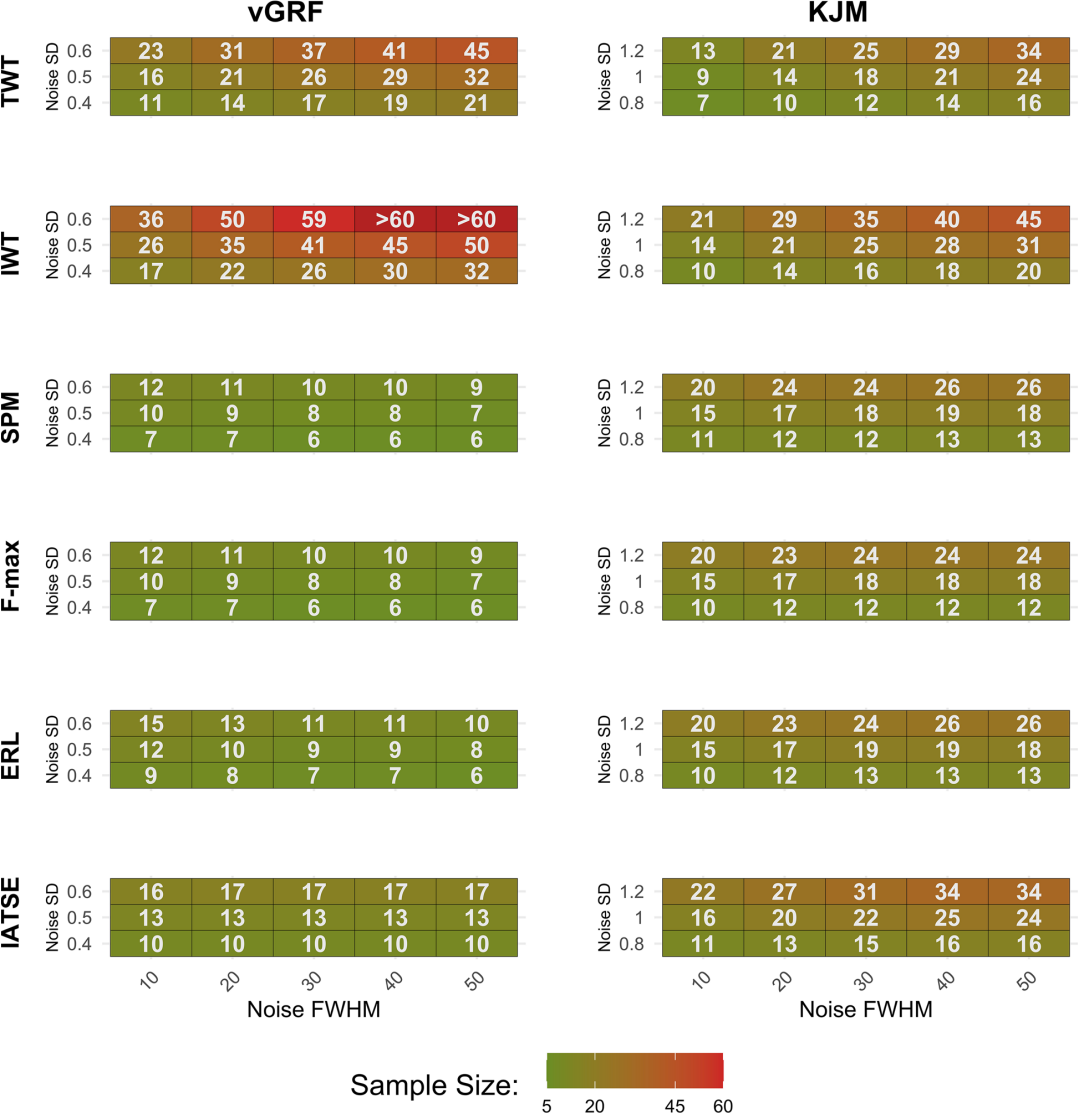
The simulation results. Noise SD and Noise FWHM represent the standard deviation of uncorrelated random Gaussian noise and noise smoothness, respectively. The reported Sample Size is the minimum sample size in each group needed to attain a statistical power of 0.80

Increasing the noise standard deviation (Noise SD) for a fixed noise smoothness level (Noise FWHM) generally increases the sample sizes required to achieve a statistical power of 0.80, regardless of the method and data characteristics (Fig. 4). For example, in vGRF data, increasing the Noise SD from 0.4 to 0.6 increases the required sample size for the F-max method from 7 to 12 when the noise smoothness level is 10. In some cases, this increase is even more considerable. For instance, the IWT method requires an increase from 26 to 59 in sample size when Noise SD increases from 0.4 to 0.6 using a noise smoothness level of 30. In contrast, for the TWT method applied to KJM data, when the Noise SD increases from 0.8 to 1 at a noise FWHM of 10, the sample size requirement changes only from 7 to 9.

When the difference between two mean functions is limited to a small portion of the domain (as illustrated by the difference function in the left panel of Fig. 1), smoother noise functions with a fixed standard deviation tend to reduce the necessary sample sizes or remain unchanged for all methods except for the TWT and IWT. For example, the SPM method requires a smaller sample size when the Noise SD is 0.5, and the sample size remains almost unchanged for the IATSE method under the same conditions (the left panel of Fig. 4). However, this trend does not hold for the TWT and IWT methods. In this case, these two methods require larger sample sizes when the noise functions become smoother because they depend on an integrated test statistic over the intervals that makes these two methods less effective for difference functions with substantial differences only in a small portion of the domain. In general, the TWT method requires smaller sample sizes compared to the IWT method. For example, with a Noise SD of 0.5 in vGRF data, the IWT method requires an increase from 26 to 50 in sample size, whereas the TWT method requires an increase from 16 to 32 when the smoothness level increases from 10 to 50.

On the other hand, when the difference function between two mean functions differ over a large portion of the domain (the right plot in Fig. 1,), smoother noise consistently leads to larger sample sizes across all methods (see the right column of Fig. 4). Although the size of this increase varies among the methods, the differences are not as prominent as when the difference is only on a small part of the domain. For instance, when the Noise SD is 0.8, the required sample sizes increase from approximately 10 to 15 for almost all methods (with the exception of the IWT method, which requires sample size of up to 20).

**Fig. 5:**
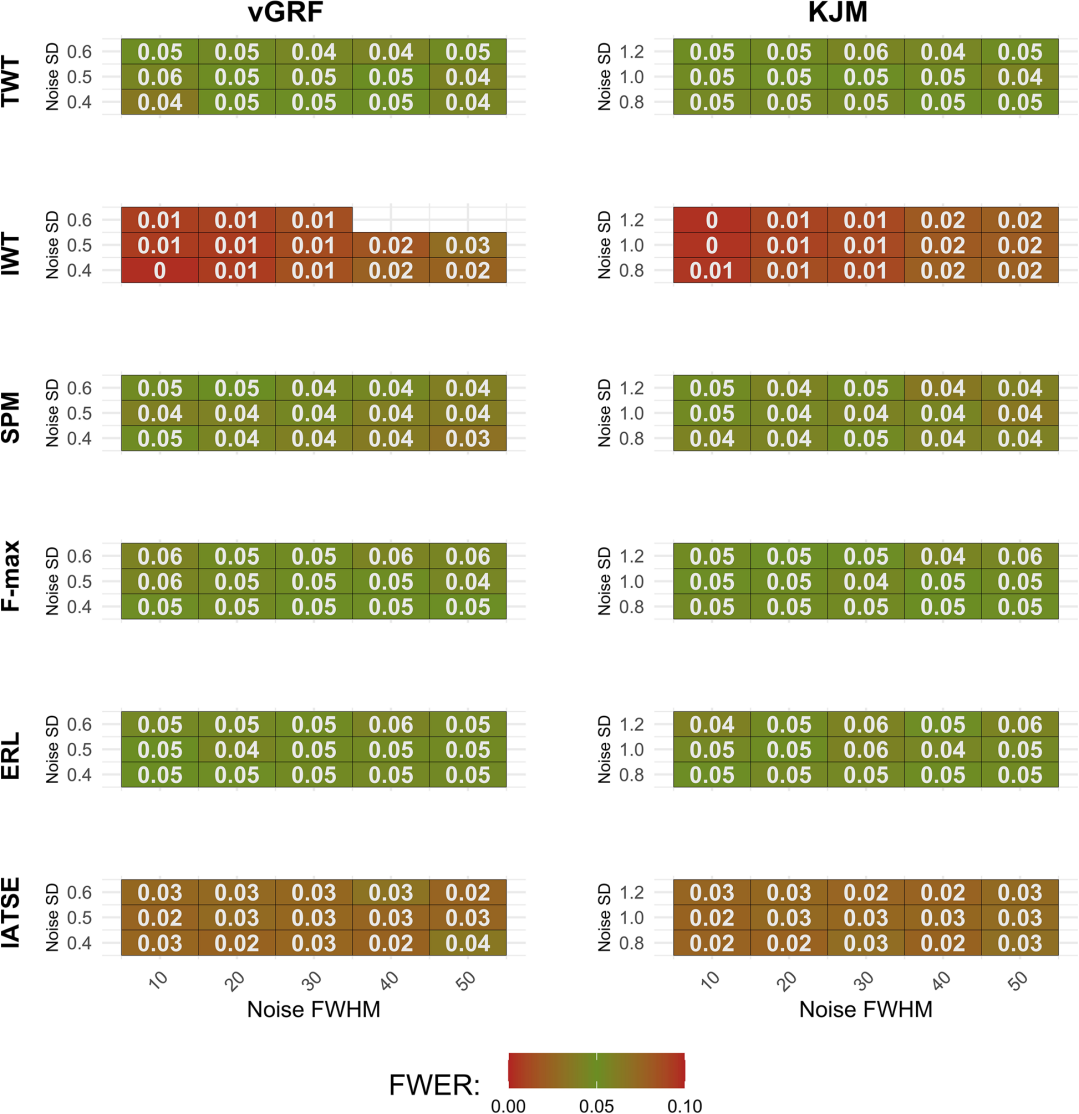
FWER assessment at the power-based sample size. Under the null hypothesis (identical mean functions, noise with specified smoothness and SD), data were simulated using the estimated sample size to achieve 0.80 statistical power in Fig. 4, and the FWER was computed over replicates. Cells are left blank for IWT when Noise SD is 0.6 and Noise FWHM values are 40 or 50, as the required sample size was only known to exceed 60

To assess another aspect of method performance beyond statistical power for a priori power analysis, we evaluated the FWER using the estimated sample sizes (Fig. 5). For example, the ERL method attains an FWER of 0.05 when the Noise SD is 0.6, the Noise FWHM is 50, and the sample size is 10 in each group. Since the required sample sizes for the IWT method with $\text {Noise SD}=0.6$ and $\text {Noise FWHM}=40$ or 50 are only known to exceed 60 (not precisely determined), the corresponding FWER entries in Fig. 5 are left blank. Although the TWT, SPM, F-max, and ERL methods tend to maintain the FWER around the significance level of $\alpha = 0.05$, the IWT and IATSE methods are more conservative. It means that they reject fewer true null hypotheses. It is important to note that the IATSE method was not originally designed to control the FWER. Moreover, the IWT method shows asymptotic control of the FWER, where smaller sample sizes result in a very low FWER (Fig. 5).

## Discussion

In this paper, we compared six well-known local methods in terms of the sample sizes required to achieve 0.80 power and examined how variations in the standard deviation and smoothness of noise functions influenced these estimates. Evaluating the performance of these methods is crucial both before data collection and during data analysis, as statistical power and the FWER are two of the key indicators of method performance.

Our primary rationale for selecting inferential methods that address local hypothesis testing is that these approaches provide explicit information about where in the domain statistically significant differences occur. Although other local inferential approaches exist, the six methods examined here are among the most widely used approaches for local hypothesis testing. This localisation is important in many applied research settings in the medical sciences, where the timing, location, or interval of a difference often carries direct scientific or clinical interpretations. For example, the onset interval of a treatment effect or a specific phase of gait is only determined by local testing. In contrast, methods that address global hypotheses evaluate equality over the entire domain and typically provide a single overall decision without identifying the particular intervals where differences arise. Nevertheless, global methods offer a coherent framework for conducting inference on entire functions, rather than aggregating multiple local decisions.

The two datasets employed in the simulation study represent two distinct effect types: one in which the substantial difference between the two mean functions spans a large portion of the domain, and another in which the difference is limited to a smaller portion. Accordingly, our simulations focused on generating data that mimics these two effect types rather than replicating actual ground reaction force or joint moment data. Two key patterns emerged regarding the sample size requirements across different methods:

1. When the part of the domain where the two mean functions have substantial differences is large, smoother data generally necessitates larger sample sizes to achieve the targeted power. Although there are some variations in the estimated sample sizes among the methods in this scenario, these differences are relatively minor. While the ERL, SPM, and F-max methods result in slightly smaller estimated sample sizes compared to the IATSE and TWT methods, the sample size estimated for IWT is slightly larger than those for the other methods.
2. When the part of the domain where the two mean functions have substantial differences is small, the trend reverses. In this case, smoother data needed fewer samples or kept the sample size almost constant for all methods except the IWT and TWT. These two methods need considerably larger sample sizes as data becomes smoother. Furthermore, the estimated sample sizes for ERL, SPM, and F-max are slightly smaller than those for IATSE.

This study has some limitations. For clarity, we only present results for a limited number of parameter values. An interested reader may, however, explore other data characteristics using the Power ShinyApp. Note that when the domain is set on the interval [0, 100], the Noise FWHM value is restricted to a maximum of 50, because larger values would exceed the domain’s capability for the smoothing process. This study focuses on balanced designs with equal sample size in each group, which are common in a priori sample size estimation. However, the inferential methods themselves are not restricted to a balanced design. In principle, they can be applied to unbalanced designs as well. Alternative simulation setups are possible, such as those based on functional principal components when pilot data are available. However, a priori sample size estimation is typically carried out at the study design stage, before the collection of any data, including pilot data. In such situations, estimates of functional principal components and error variance are typically unavailable, and sample size calculations must instead rely on prior knowledge or guesses about the potential data.

Many practical studies cannot collect perfectly balanced groups. Consequently, exploring power analysis under unequal sample sizes per group for functional hypotheses would be valuable. In addition, real-world data might exhibit non-stationary noise variance across the domain. Investigating how such heteroscedasticity impacts power and how to adjust sample-size recommendations to maintain desired power levels is an interesting direction for future work.

## Conclusions

Our findings emphasise the importance of performing a priori sample size estimation and selecting analysis methods that align with the characteristics of the collected data to ensure valid inference. The Power ShinyApp not only facilitates a priori power analysis by providing various options for data selection and inferential methods, but also allows users to explore how changes in parameter values affect the data and the statistical power.

It is important to note that the choice of an inferential method should not be guided solely by which approach determined the smallest required sample size. Instead, method selection should be primarily informed by the scientific question and specific hypotheses of interest. It ensures that the inferential method aligns appropriately with the study objectives. If you already have collected data, and want to choose among the inferential methods, be aware that data characteristics such as noise smoothness, noise standard deviation, and the mean function differences affect methods very differently. For example, with the same anticipated data characteristics, one approach might need only 10 subjects per group to reach adequate power, whereas another could require 60 for valid inference.

## Recommendations

In order to do a priori sample size estimation, we propose the following step-by-step framework of recommendations:

1. Specify the scientific question and the statistical hypothesis.
2. Decide on the inferential method you intend to use for data analysis. Different methods have different statistical properties and may rely on distinct assumptions. Keep in mind that in many medical studies, controlling error rates (FWER or FDR) is also important.
3. Clearly state the definition of statistical power used and the target power level. A typical target level is 0.80, but some studies may justify higher or lower levels.
4. Use pilot data to estimate noise standard deviation and smoothness, or explore potential parameter values based on the characteristics you expect to observe after data collection.
5. Calculate the sample size required to achieve the target power level.
6. Report your statistical power calculations in study protocols and ethics submissions. We recommend documenting the anticipated mean functions, the inferential method, and the values of all parameters used in the power analysis that informed the sample size calculation.

Steps 1–5 describe what should be done before the data collection takes place, while the last step describes what should be reported. These recommendations, together with the easy-to-use interactive application, simplify for researchers working with a priori power analysis for functional (curve) data, from study design to final reporting. This makes the statistical plan explicit and reproducible and helps reviewers and others assess study feasibility.

## Supplementary Information

Supplementary Material 1.

## Data Availability

The reproducible code is available at: https://github.com/mr-seydi/SampleSize_FDA. All simulations were based on publicly available data cited in the manuscript.
