# Supplementary material for: Sample size estimation for local hypothesis testing of functional data in medical studies: method comparison, recommendations, and a web application
Source: BMC Med Res Methodol. 2026 Jan 22;26:19. doi: 10.1186/s12874-026-02772-w (PMC12853631; doi:10.1186/s12874-026-02772-w)
Supplement: Supplementary file 1 — Supplementary Material 1. [file 12874_2026_2772_MOESM1_ESM.pdf]

Appendix A: Functional Hypotheses

For functional data, there are generally two approaches for conducting a hypothesis test: a global hypothesis test

$$\begin{cases} H_0 : \mu_1(t) = \mu_2(t), & \text{for almost every } t \in \mathcal{D} \\ H_1 : \mu_1(t) \neq \mu_2(t), & \text{on a set of non-zero measure in } \mathcal{D} \end{cases}$$

where equality $\mu_1 = \mu_2$ is defined in the Lebesgue sense and the local hypothesis test as it is defined in the body of the paper. In contrast to the local methods, the global methods, which address the global hypothesis test, may not indicate where within the domain significant differences are detected. For example, integral-based approaches using $\mathcal{L}^p$ distances typically provide only an overall measure of difference without localising the effect.

Appendix B: Methods

The aim of this appendix is to clarify, step by step, how each method is practically implemented within our simulation framework to estimate statistical power. In this study, an unadjusted p-value function refers to p-values without any correction for multiple testing. In this context, the multiple testing issue is over the discretisation of the domain. In practice, all implementations are based on the discretisation of the domain D. In contrast, an adjusted p-value function provides corrected values by applying methods designed to address the simultaneous multiple-testing problem. When multiple tests are simultaneously conducted, an inference goal is often to maintain the family-wise error rate (FWER) at a level of α.

B.1 Statistical Parametric Mapping (SPM)

The adjusted p-value using the SPM method is calculated by:

1. For all finite number of $t \in \mathcal{D}$, evaluate the pointwise test statistic $T^*(t)$.
2. Define T^*_M as the random variable corresponding to the maximum test statistic. Then, for all $t \in \mathcal{D}$ determine

   $$P\Big(T^*_M \geq T^*(t)\Big) \approx \rho_0\Big(T^*(t)\Big) + \mathcal{R}\,\rho_1\Big(T^*(t)\Big),$$

   where ρ_0 and ρ_1 are 0 and 1-dimensional EC densities, respectively (Worsley et al., 1996). The number of Resels (resolution elements), $\mathcal{R}$, is defined as (Q-1)/FWHM, where Q is the number of nodes (evaluated points in the domain) and FWHM denotes estimated noise smoothness.
3. Estimate the adjusted p-value function as

   $$\forall t \in \mathcal{D}, \quad \hat{p}_{\text{SPM}}(t) = 1 - exp\bigg(-P\Big(T^*_M \geq T^*(t)\Big)\bigg).$$

Note that EC densities are valid only for high threshold values, corresponding to low p-values (typically p-value < 0.5). As a result, this approach does not generate accurate p-value functions in general, particularly for larger p-values. However, since this study focuses on rejection decisions, which correspond to small p-values (< 0.05), the use of EC densities remains appropriate within their valid range.

Resels quantify the smoothness of the data and are used to appropriately adjust the EC densities (Worsley, 1994). The FWHM function is estimated using $\text{FWHM}(t) = \sqrt{4 \log 2/\lambda(t)}$, where $\lambda(t)$ represents the estimated gradient normalised by the residual sum of squares. The overall FWHM utilised in the Resels calculation is then obtained by integrating $\text{FWHM}(t)$ over time.

This approach ensures strong control of the FWER. SPM framework can be easily extended to different test statistic by appropriately modifying the EC densities.

B.2 F-max

There are two different versions of this local test, the single-threshold test and the step-down procedure.

The adjusted p-value for the single threshold test is calculated by:

1. For all finite number of $t \in \mathcal{D}$, evaluate the pointwise test statistic $T^*(t)$.
2. Estimate the test statistic $T^{*b}(t)$ for all $t \in \mathcal{D}$, and $b = 1, \ldots, B$ random permutation.
3. Define T_M^{*b}, the maximum of the test statistic $T^{*b}(t)$ across $t \in \mathcal{D}$ for each $b \in \{1, \ldots, B\}$.
4. Estimate the adjusted p-value function as

$$\forall t \in \mathcal{D}, \quad \hat{p}_{\text{F-max}}(t) = \frac{1}{B} \sum_{b=1}^{B} \mathbb{I}\Big(T_M^{*b} \geq T^*(t)\Big) \tag{1}$$

The adjusted p-value for the step-down method is calculated by:

1. Start with the same first three steps as the single threshold test.
2. Identify the largest test statistic $T^*(t)$ over all $t \in \mathcal{D}$ and calculate the adjusted p-value for it using the equation (1).
3. Remove that point and continue this process until all points have been tested and removed.

Both versions provide strong control of the FWER. However, the step-down version is computationally expensive. The single threshold procedure is commonly used in biomechanics applications. This is demonstrated by Pataky et al. (2022), Pataky et al. (2021), and Warmenhoven et al. (2018). Consequently, it is the version used in this paper.

B.3 Interval-wise Testing (IWT)

The IWT's adjusted p-value provides interval-wise control of the FWER. Calculating the adjusted p-value in IWT involves the following steps:

1. Construct the family $\mathcal{F}$ consisting of all intervals of the domain $\{[t_1, t_2] \mid t_1, t_2 \in \mathcal{D} \text{ and } t_1 < t_2\}$.
2. Approximate a test statistic $T^{\mathcal{I}}$ based on the $\mathcal{L}^2$ distance for each interval $\mathcal{I} \in \mathcal{F}$ by

$$T^{\mathcal{I}} = \frac{\|\hat{\mu}_1^{\mathcal{I}}(t) - \hat{\mu}_2^{\mathcal{I}}(t)\|_{\mathcal{L}^2}^2}{|\mathcal{I}|} = \frac{1}{|\mathcal{I}|} \int_{\mathcal{I}} \Big(\hat{\mu}_1^{\mathcal{I}}(t) - \hat{\mu}_2^{\mathcal{I}}(t)\Big)^2 \, dt, \tag{2}$$

   where $|\mathcal{I}|$ represents the Lebesgue measure of the interval $\mathcal{I}$.
3. Using B random permutations, estimate the p-value $p^{\mathcal{I}}$ for the test $H_0^{\mathcal{I}}$ vs. $H_1^{\mathcal{I}}$ for each interval $\mathcal{I} \in \mathcal{F}$.
4. Estimate the adjusted p-value function as

$$\hat{p}_{\text{IWT}}(t) = \sup_{\mathcal{I} \in \mathcal{F} \text{ s.t. } t \in \mathcal{I}} p^{\mathcal{I}}.$$

Since this method is based on all intervals of the domain, as described earlier, it is computationally intensive.

B.4 Threshold-wise Testing (TWT)

This method makes corrections on the unadjusted p-value function and does not use a predefined family, therefore, it is a data-dependent technique. In TWT, the adjusted p-value is calculated as follows:

1. Define the set $\{\mathcal{Z}_j\}_{j=1}^J$ containing J equal-sized intervals obtained by partitioning the domain $\mathcal{D}$ such that $\bigcup_{j=1}^J \mathcal{Z}_j = \mathcal{D}$, $\mathcal{Z}_j \cap \mathcal{Z}_{j'} = \varnothing$ for all $j \neq j'$, and $\mathcal{Z}_j \subseteq \mathcal{D}$.
2. Approximate the test statistic $T^{\mathcal{Z}_j}$ for each interval $j = 1, \ldots, J$, as introduced in the equation (2).
3. Estimate the unadjusted p-value $p^{\mathcal{Z}_j}$ for all $j = 1, \ldots, J$ using B random permutations.
4. Define a finite and discretised set of thresholds $\Theta = \{\theta\}$ over the interval [0, 1]. For each threshold, define

$$\mathcal{S}^{\geq \theta} = \{\mathcal{Z}_j \mid j = 1, \ldots, J \text{ and } p^{\mathcal{Z}_j} \geq \theta\}$$

$$\mathcal{S}^{< \theta} = \{\mathcal{Z}_j \mid j = 1, \ldots, J \text{ and } p^{\mathcal{Z}_j} < \theta\}.$$

5. Compute the p-values for the two sets, $p_{\geq\theta}$ and $p_{<\theta}$, based on the combined test statistic. Define a new p-value function as

$$p^{\theta} = \begin{cases} p_{\geq\theta} & \text{if} \quad \mathcal{Z}_j \in \mathcal{S}^{\geq\theta} \\ p_{<\theta} & \text{if} \quad \mathcal{Z}_j \in \mathcal{S}^{<\theta} \end{cases}$$

6. Estimate the adjusted p-value function as

$$\forall t \in \mathcal{Z}_j, \quad \hat{p}_{\text{TWT}}(t) = \max_{\theta\in\Theta} p^{\theta}$$

Since Θ is defined as a finite set and $\mathcal{F}$ in IWT is an infinite set, the max is used here instead of sup. The TWT has a weak control of the FWER for large B.

B.5 Extreme Rank Length (ERL) Envelope

The lower $C^{\alpha}_{low\ t}$ and upper $C^{\alpha}_{upp\ t}$, $100(1-\alpha)\%$ simultaneous confidence bands mean that

$$P(\delta^{*b}(t) \notin [C^{\alpha}_{low\ t},\, C^{\alpha}_{upp\ t}] \quad \text{for any} \quad t \in \mathcal{D}) = \alpha,$$

where $\delta^{*b}(t)$ determines the test statistic for all $t \in \mathcal{D}$, and $b = 1, \ldots, B$ random permutations. A set of $(C^{\alpha}_{low},\, C^{\alpha}_{upp})$ represents the global envelope which is defined w.r.t O_b, the order of curve δ^{*b}, if

- $O_{(\alpha)} \in \mathbb{R}$ is the largest O_b such that the number of b for which $O_b < O_{(\alpha)}$ is less or equal to αB.
- $\delta^{*b}(t) < C^{\alpha}_{low\ t}$ or $\delta^{*b}(t) > C^{\alpha}_{upp\ t}$ for some $t \in \mathcal{D}$ iff $O_b < O_{(\alpha)}$ for every permutations.
- $\forall t \in \mathcal{D} \quad C^{\alpha}_{low\ t} \leq \delta^{*b}(t) \leq C^{\alpha}_{upp\ t}$ iff $O_b \geq O_{(\alpha)}$ for every $t \in \mathcal{D}$.

The curves are ordered so that the smaller O_b belongs to the most extreme δ^{*b}. The functional hypothesis test of interest can be conducted using the ERL method by:

1. For all finite numbers of $t \in \mathcal{D}$, evaluate the test statistic of the observed data, $\delta^{*}(t)$.
2. Estimate the test statistic $\delta^{*b}(t)$ for all $t \in \mathcal{D}$, and $b = 1, \ldots, B$ random permutations.
3. Consider $o_b(t)$ as the raw rank of $\delta^{*b}(t)$ for fixed t, if there are ties raw ranks are averaged. The pointwise rank is calculated as $O_b(t) = \min(o_b(t), B + 1 - o_b(t))$ for two-sided case.

4. O_b is defined by arranging the vector of $O_b(t)$ for $t \in \mathcal{D}$ from smallest to largest. Afterwards, calculate

$$E_b = \frac{1}{B}\sum_{b'=1}^{B} I(O_{b'} \prec O_b)$$

where

$$O_{b'} \prec O_b \Longleftrightarrow \exists n^* \in \mathcal{D} \quad O_{b'}(t) = O_b(t) \quad \forall t < n^*, \quad O_{b'}(n^*) < O_b(n^*).$$

5. Determine $O_{(\alpha)}$ and the index set $I_\alpha = \{b \in 1, \ldots, B : E_b \geq O_{(\alpha)}\}$. The extreme rank length envelope is defined as

$$C^{\alpha}_{low\ t} = \min_{b \in I_\alpha} \delta^{*b}(t) \quad \text{and} \quad C^{\alpha}_{upp\ t} = \max_{b \in I_\alpha} \delta^{*b}(t) \quad \text{for} \quad t \in \mathcal{D}.$$

6. If the observed test statistic function falls outside the envelope at any point in the domain, the null hypothesis is rejected at the given significance level.

The extreme rank length is one of the global envelope methods in which curves are ordered so that curves with longer lengths of pointwise extreme ranks are deemed more extreme. This method provides weak control over FWER (Mrkvička and Myllymäki, 2024). For a full description of this method, see Mrkvicka et al. (2016).

B.6 Iterative Adaptive Two-stage Envelope (IATSE)

This method controls the false discovery rate (FDR) in functional hypothesis testing. The hypothesis is rejected if the test statistic is outside of the envelope that is constructed as

1. For all N_0 values of $t \in \mathcal{D}$, evaluate the pointwise test statistic $T(t)$.

2. Estimate the test statistic $T^b(t)$ for all $t \in \mathcal{D}$, and $b = 1, \ldots, B$ random permutations.

3. Find the rejection region by constructing a min-max envelope

$$C^{\gamma}_{\min\ t} = \overset{\gamma}{\min_{b=1,\ldots,B}} T^b(t) \quad \text{and} \quad C^{\gamma}_{\max\ t} = \overset{\gamma}{\max_{b=1,\ldots,B}} T^b(t) \quad \text{for} \quad t \in \mathcal{D},$$

where $\gamma = 1, \ldots, [B/2]$ denotes the rejection threshold, $C^{\gamma}_{\min\ t}$ and $C^{\gamma}_{\max\ t}$ are the γ-th smallest and largest values of the test statistic.

4. Find the largest rejection region such that

$$\frac{E(\mathrm{FP})}{\max(\mathrm{FP}+\mathrm{TP}, 1)} = \frac{2N_0 \frac{\gamma}{B}}{\max(\mathrm{FP}+\mathrm{TP}, 1)} \leq \alpha,$$

where FP and TP indicate false and true positives, respectively. If the largest rejection region (FP + TP) is equal to zero, STOP and choose γ for the envelope. Otherwise, go to the next step.

5. Estimate
$$\hat{\pi}_0 = \min\Big(1, \frac{N_0 - (\mathrm{FP} + \mathrm{TP})}{N_0(1 - \frac{2\gamma}{B})}\Big).$$
6. Find the largest rejection region such that
$$\hat{\pi}_0 \frac{2N_0 \frac{\gamma}{B}}{\max(\mathrm{FP} + \mathrm{TP}, 1)} \leq \alpha.$$
7. The null hypothesis is rejected at the chosen significance level if the observed test statistic function exceeds the envelope at any point within the domain.

If γ in the third step is a non-integer value, the envelope needs to be modified (Mrkvička and Myllymäki, 2023). For a fixed γ, only the number of rejection points, $\mathrm{FP} + \mathrm{TP}$ can be observed while the number of FP is not observable but can be estimated

$$\mathrm{FDR} = E(\frac{\mathrm{FP}}{\mathrm{FP} + \mathrm{TP}}) \approx \pi_0 \frac{E(\mathrm{FP})}{\max(\mathrm{FP} + \mathrm{TP}, 1)}.$$

Under the null, each test has the same chance of being rejected, the rank of the observed test statistic at any given point is uniformly distributed among B permutations. For a two-sided test, rejections occur if the observed statistic falls below or above the upper envelope. Under the null, the probability that a single test statistic is more extreme than the envelope defined by γ is approximately γ/B. Since there are N_0 tests, the expected number of false rejections is $N_0\gamma/B$. When accounting for the two-sided case, you multiply by 2. Therefore, $E(\mathrm{FP}) = 2N_0\gamma/B$.

Appendix C: Methods Performance

Time performance was assessed on a 2020 MacBook Pro 13" (Apple M1, 16GB unified memory) using R version 4.4.1. Results are reported for three sample sizes: 10, 30, and 50 over 20 repetitions. Note that time is based on the current implementation and may vary with new implementations, different software or hardware setups.

Table C.1: Timing summary for Sample Size = 10. All times are in **milliseconds**.

**Method**	**Min**	**LQ**	**Mean**	**Median**	**UQ**	**Max**
SPM	5.12	5.26	7.20	5.34	5.39	39.66
F-max	248.72	251.09	265.93	254.47	259.12	397.41
IWT	1505.55	1600.10	1647.34	1651.45	1706.32	1776.03
TWT	62.67	64.93	69.18	65.88	72.78	99.91
ERL	83.58	84.49	88.66	85.25	86.35	125.07
IATSE	112.27	116.04	127.88	121.67	124.64	252.81

Abbreviations: **Min** = minimum runtime; **LQ** = lower quartile (25th percentile); **Mean** = average runtime; **Median** = 50th percentile; **UQ** = upper quartile (75th percentile); **Max** = maximum runtime.

Table C.2: Timing summary for Sample Size = 30. All times are in **milliseconds**.

**Method**	**Min**	**LQ**	**Mean**	**Median**	**UQ**	**Max**
SPM	5.45	5.48	5.63	5.54	5.59	6.31
F-max	637.55	649.27	686.86	656.77	727.60	792.86
IWT	1539.08	1597.97	1665.13	1663.37	1718.38	1886.60
TWT	79.36	87.25	90.11	89.83	94.48	97.28
ERL	94.03	100.16	115.44	101.12	104.61	243.99
IATSE	154.63	160.98	166.37	164.20	168.01	203.92

Abbreviations: **Min** = minimum runtime; **LQ** = lower quartile (25th percentile); **Mean** = average runtime; **Median** = 50th percentile; **UQ** = upper quartile (75th percentile); **Max** = maximum runtime.

Table C.3: Timing summary for Sample Size = 50. All times are in **milliseconds**.

**Method**	**Min**	**LQ**	**Mean**	**Median**	**UQ**	**Max**
SPM	5.54	5.69	5.81	5.78	5.81	6.99
F-max	987.21	1022.22	1083.24	1044.94	1142.69	1222.26
IWT	1563.91	1685.53	1746.10	1753.86	1785.56	1972.65
TWT	109.93	117.09	122.76	120.82	125.47	164.42
ERL	110.03	115.19	126.56	119.83	122.59	260.95
IATSE	211.00	222.11	239.24	224.97	237.60	390.50

Abbreviations: **Min** = minimum runtime; **LQ** = lower quartile (25th percentile); **Mean** = average runtime; **Median** = 50th percentile; **UQ** = upper quartile (75th percentile); **Max** = maximum runtime.

References

Mrkvička, T. and Myllymäki, M. (2023). False discovery rate envelopes. *Statistics and Computing*, 33(5):109.

Mrkvička, T. and Myllymäki, M. (2024). Comparison of approaches for local testing with functional test statistics. *Journal of Statistical Computation and Simulation*, pages 1–18.

Mrkvicka, T., Myllymaki, M., Jilek, M., and Hahn, U. (2016). A one-way anova test for functional data with graphical interpretation. *arXiv preprint arXiv:1612.03608*.

Pataky, T. C., Abramowicz, K., Liebl, D., Pini, A., de Luna, S. S., and Schelin, L. (2021). Simultaneous inference for functional data in sports biomechanics: Comparing statistical parametric mapping with interval-wise testing. *AStA Advances in Statistical Analysis*, pages 1–24.

Pataky, T. C., Robinson, M. A., Vanrenterghem, J., and Donnelly, C. J. (2022). Simultaneously assessing amplitude and temporal effects in biomechanical trajectories using nonlinear registration and statistical nonparametric mapping. *Journal of Biomechanics*, 136:111049.

Warmenhoven, J., Harrison, A., Robinson, M. A., Vanrenterghem, J., Bargary, N., Smith, R., Cobley, S., Draper, C., Donnelly, C., and Pataky, T. (2018). A force profile analysis comparison between functional data analysis, statistical parametric mapping and statistical non-parametric mapping in on-water single sculling. *Journal of Science and Medicine in Sport*, 21(10):1100–1105.

Worsley, K. J. (1994). Local maxima and the expected euler characteristic of excursion sets of χ 2, f and t fields. *Advances in Applied Probability*, 26(1):13–42.

Worsley, K. J., Marrett, S., Neelin, P., Vandal, A. C., Friston, K. J., and Evans, A. C. (1996). A unified statistical approach for determining significant signals in images of cerebral activation. *Human brain mapping*, 4(1):58–73.
